# The influence of valence shifts in fear appeals on message processing and behavioral intentions: A moderated mediation model

**DOI:** 10.1371/journal.pone.0255113

**Published:** 2021-09-02

**Authors:** Perina Siegenthaler, Alexander Ort, Andreas Fahr

**Affiliations:** 1 Department of Communication and Media Research, University of Fribourg, Fribourg, Switzerland; 2 Department of Health Sciences and Medicine, University of Lucerne, Lucerne, Switzerland; University of Connecticut, UNITED STATES

## Abstract

Newer approaches in health communication research indicate that understanding the flow of emotional experiences during exposure to fear appeals can clarify their persuasive effects. In a laboratory experiment, the impact of valence shifts during exposure to fear appeals on determinants of health-relevant behaviors were examined. Continuous response measurement allowed gathering real-time data about participants’ experiences of valence shifts during exposure. Among the results, a shift from negative to positive valence promoted efficacy perceptions but only for people being personally affected by the health issue. Perceived efficacy, in turn, increased intentions to put recommended behaviors into practice. This suggests that inducing positive valence shifts in health messages improves their effectiveness, especially for relevant target groups.

## Introduction

Researchers in health communication—most recently, Moyer-Gusé et al. [1], Nabi and Myrick [2], and Xu and Guo [3], among others—have put a lot of effort in examining the persuasive effects of various emotional appeals on determinants of health-relevant behaviors. Among their findings, messages concerning health issues that contain emotional appeals foster information processing, goal commitment, and goal attainment and, ultimately, promote personal health [4]. To that end, messages may, for example, illustrate the negative consequences of unhealthy behaviors or spotlight the rewards of healthy ones and/or ways to achieve those benefits [5]. Against that background, researchers have also extensively investigated the effects of different emotional appeals, not only fear [6] but also disgust [7, 8], humor [9], hope [10], and guilt [11, 12].

From another angle, the assumption that each mediated health-message evokes only one emotion has been challenged [e.g., 13, 14]. Critics have highlighted that emotions are not only fleeting but also assumed to underlie transformations during media exposure that necessarily result from continuous evaluations and reappraisals of the content of messages and personal experiences [4, 15, 16]. For those reasons, people often experience different emotions while being exposed to messages, whether those emotions cascade one after another over time [17] or arise together at the same time for so-called “mixed emotions” [18]. According to that logic, fear appeals, as a predominant strategy in health communication, usually involve not only threat-evoking but also efficacy-fostering elements. Whereas the threatening information is presumably associated with the experience of fear, subsequent efficacy cues may cause a shift from fear to another emotion such as hope [14]. That particular evolution of shifts in emotional experiences is likely to influence the processing and effectiveness of mediated information in non-arbitrary ways. Drawing from such knowledge, recent investigations into the processing and effects of mediated messages have emphasized the importance of considering the dynamic of underlying emotional experiences during exposure to such messages, or what has been termed emotional flow [14]. Similar approaches have referred to changes in emotional experiences as emotional trajectories [e.g., 13], based on the assumption that such experiences stem from the interplay of the activation of the appetitive and aversive motivational systems.

Although neatly laid out in theory, the occurrence, development, and consequences of the evolution of emotional experiences have thus far received little empirical examination, save from Peinado [19] and other authors using similar approaches [e.g., 13, 20]. In response, we sought to prove, describe, and explain the effects of changes in emotional experiences during exposure to mediated health messages that follow the popular threat-then-efficacy pattern of fear appeals. While tracking that pattern, we investigated the impact of shifts in valence, namely from negative to positive, on message processing and on important determinants of health-related behavior. As a result, our study contributes to an emerging conceptual understanding of emotional flows and sets the stage for further examinations of that process-oriented perspective in research on health communication.

## Changes in emotional experiences during exposure to fear appeals

Emotional appeals play an important role in persuasive health communication, primarily because they are assumed to generate certain advantages for information processing over the sober presentation of facts. Emotional appeals are assumed to increase attention [21], for example, as well as be more memorable [22] and foster attitude [23] and/or behavior change [6]. The importance of emotional appeals is also reflected in researchers’ significant investments of resources into understanding the role of emotions in the effects of messages [e.g., 5, 15, 23–28]. Indeed, numerous theories about fear appeals—for instance, Rogers’s [29] protection motivation theory and Witte’s [5] extended parallel process model (EPPM)—either incorporate emotionally dependent responses (e.g., threat appraisals) as crucial factors or directly account for emotional experiences with reference to them. Because those models are designed to probe the effects of fear appeals, they often exclusively focus on that particular emotional experience as a desirable response to the information presented [23]. The strategy behind such appeals entails triggering an emotional reaction—fear—by threatening target audiences with the harmful outcomes of particular unhealthy behaviors. As a consequence, the appeals intend to elicit adaptive reactions from audiences by motivating them to alter their behavior in accordance with the recommendations [30].

Observed in that light, fear appeals tend to follow a threat-then-coping pattern [5], in which initial threatening information is followed by information about or recommendations for avoiding the threat. According to the EPPM [5] and the outlined structure of fear appeals, after initially assessing the threat (i.e., its severity and personal susceptibility to it), audiences evaluate their ability to act, i.e., self-efficacy, and their personal belief about the recommended behavior’s effectiveness, i.e., response efficacy. Extensive investigations into the effects of the threat-then-coping pattern have confirmed its persuasive potential, especially compared with messages containing only threats or only efficacy cues [31–33]. The emotional experience of fear as a result of evaluating personal relevance (i.e., susceptibility) and the significance (i.e., severity) of the health risk presented has also demonstrated its vital role in message processing. Even so, fear appeals can be expected to evoke not only fear during exposure to such messages. After all, changes in the nature of the information provided suggest that audiences’ emotional experiences shift from fear to other emotions, largely because, in subsequent evaluations, people assess their potential to cope with the risk (i.e., response efficacy and self-efficacy). Thus, while being exposed to and processing messages according to the conventional pattern of fear appeals, individuals are likely to experience not one but various emotional reactions.

Nabi [14] has addressed the dynamics resulting from various types of message cues and their impacts on emotional experiences by introducing the concept of emotional flow (cf. emotional trajectory [13]), defined as “the evolution of the emotional experience during exposure to a health message, marked by one or more emotional shifts” (p. 117). The occurrences of those shifts are manifold and can assume several shapes and characteristics. As such, the shifts can manifest as changes in valence from negative to positive emotional states and vice versa (e.g., fear to happiness or pride to sadness) and even evolve within the same valence (e.g., fear to anger or happiness to pride). Emotional shifts thus refer not only to shifts between discrete emotions but also include shifts in valence. Beyond that, shifts in the intensity of the same emotional state can also unfold (e.g., slightly to heavily disgusted). If shifts occur during exposure to a message, then which and how many of them occur depends upon the message’s structure and design in interaction with the individual’s appraisals.

Changes in emotional experiences (i.e., emotional or valence shifts) presumably benefit the act of information processing and the outcomes of persuasive messages for several reasons. For one, exposure to messages evoking negative emotions that, in turn, elicit strong negative emotional experiences results in poor information processing and can even trigger defensive responses [e.g., 34]. For another, based on the notion of mixed emotions, Mukherjee and Dubé [35] found that adding humor to fear appeals reduces defensive responses, which in turn increases their persuasiveness. Taken together, the occurrence of positively and negatively valenced emotions may prevent overarousal as well as defensive responses, as supported by the notion that positive emotions buffer and facilitate the processing of information about a risk or threat [21, 36]. In further support, researchers have even demonstrated that fear appeals with a pronounced so-called “happy ending” reduce the onset of defensive responses [20]. On top of that, Rossiter and Thornton [37] found that relief from momentum at the end of a message improved the effectiveness of a fear appeal in terms of the performance of the recommended behavior. Thus, closely examining shifts from fear to positive emotions seems worthwhile, for they might be particularly beneficial and effective in the context of information processing and persuasiveness of health-related messages.

In sum, messages that succeed in effecting change by first promoting a threat and by later fostering efficacy perceptions and positive emotional experiences are presumably effective in supporting important determinants of adaptive health-relevant behaviors. However, research on persuasive (fear) appeals in health communication has rarely addressed the evolving nature of emotional experiences during exposure to messages as a crucial factor influencing the processing and outcomes of such messages (for exceptions, see [2, 14, 19, 38]). Therefore, the purposes of our study were to induce emotional flows, measure the valence shifts experienced, and examine their effects on central determinants of health-relevant information processing and behavior. As this study focuses on the effects of valence shifts (no shift vs. shift) and not on the effects of discrete emotions on health-relevant outcomes, we will use the term “valence shift” instead of “emotional shift” in the following.

## Research interest and hypotheses

As outlined above, emotional flows are induced by a message’s structure, and shifts can occur over the course of media exposure. By extension, our research involved examining the effects of shifts from a negative valence (fear) to either another negative or a positive valence, which follows the basic understanding of fear appeals and their conventional threat-then-efficacy pattern.

**Fig 1:**
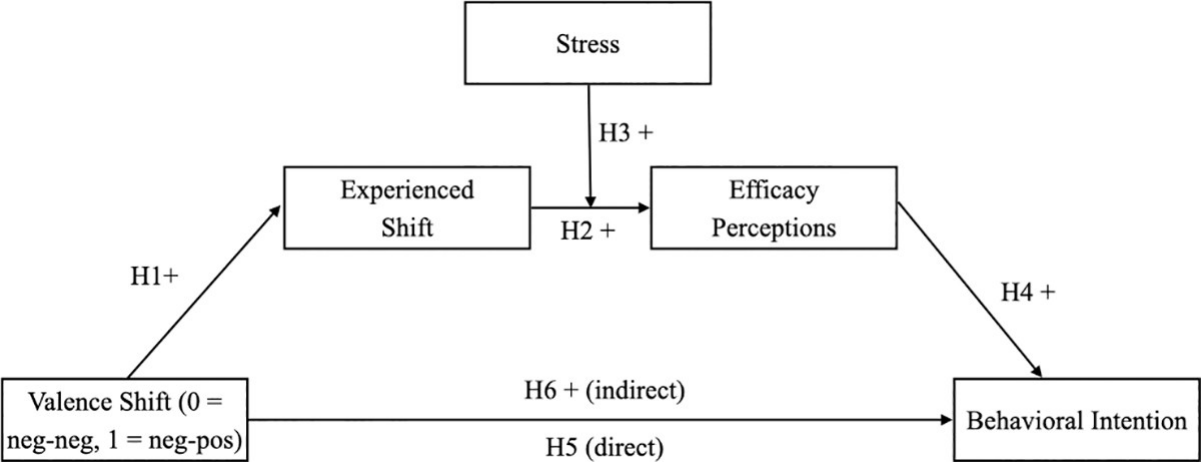
Effects of valence shift on behavioral intention mediated by experienced shift and efficacy perceptions and moderated by stress.

Several hypotheses guided our investigation. We expected that exposure to a health-related fear appeal with a shift in valence from negative (i.e., fear) to positive strengthens the experience of a positive shift relative to messages containing shifts within the same valence (H1 [19]). In accordance with the EPPM [5] as a theoretically and empirically well-established model of how fear appeals are processed, as well as the concept of emotional flow [14], we also hypothesized five other relationships. For one, individuals experiencing a positive shift (i.e., from negative to positive valence) during exposure to a message are expected to have higher efficacy perceptions than individuals without said experience (H2). For another, because being affected by a health issue is an important moderating factor that exerts decisive influence on the effectiveness of fear appeals [e.g., 22, 39–41], we assumed that being affected by the health threat presented positively moderates the relationship between the experienced shift and efficacy perceptions (H3). Following the EPPM, we also hypothesized a positive relationship between perceived efficacy and behavioral intentions (H4). In contrast to direct stimulus-response models, many theories and models in persuasive health communication [e.g., 5, 29] suggest that cognitive and emotional processes act as intermediary between the message and the outcome and are crucial prerequisites for health messages to be effective. Therefore, we expected the fear appeal to impact behavioral intentions not directly (H5) but with an indirect, positive effect via the valence shift experienced and perceived efficacy (H6), all assuming that participants are affected by the topic (see H3). Fig 1 depicts the hypothesized relationships.

## Method

### Research design and procedure

To investigate the effects of valence shifts in messages following the pattern of fear appeals (i.e., threat-then-efficacy information), we employed a single-factorial, between-subjects experimental design. A week before the experiment, an online pre-questionnaire was administered to collect participants’ sociodemographic data and information about their personality traits. Prior to the experiment, participants arrived at the laboratory, signed their informed consent, and were randomly assigned to one of the two experimental groups. Next, participants watched the video in a cinema-like environment. For the stimulus, short video clips about work-related stress were manipulated to include shifts from a threat (i.e., inducing fear for a negative valence) to five different emotions (i.e., triggering shifts or no shifts in valence). As this analysis focuses on the effects of valence shifts (no shift vs. shift) and not on the effects of distinct emotions, we used five different emotions to induce the valence shift to ensure that the effect of the valence shift can clearly be attributed to valence and not to a particular discrete emotion. The five groups were collapsed in two types of flows for analysis, i.e., from negative to negative valence (no shift) versus negative to positive valence (shift). Concerning valence and following theoretical work on emotions, fear, anger, and disgust conditions were collapsed into the no-shift group, while hope and pride conditions were merged into one shift group. To capture the dynamic flow of emotional experiences and to measure shifts in valence during exposure to the message, we used continuous response measurement (CRM). Participants were given a tablet and instructed to continuously indicate their personal experience while watching the video in terms of valence. More specifically, they were told to use the slider on the tablet to indicate their current emotional state, that is, how pleasant or unpleasant they feel at each moment. In order to indicate a very pleasant feeling, participants could move the slider all the way up. For a very unpleasant feeling, they could move the slider all the way down. In between, participants could gradually indicate their feelings. Data were gathered using the software solution from https://www.real-time-response.de/?lang=en [42]. After exposure, participants responded to a second questionnaire examining the effects of the different manipulations on the EPPM’s central factors regarding message processing and behavioral intentions. After completing the questionnaire, participants received compensation equivalent to US $10. This work was approved (written consent) by the “Commission cantonale d’éthique de la recherche” of the canton of Bern, Switzerland (Project #2017–00447).

### Sample

A total of 252 participants were recruited on campus at a university in Switzerland. Due to missing data, the characteristics of the sample are based on a total of 243 cases with a mean age of 23.3 (*SD* = 2.54). Of the respondents, 69% were women and most participants were students (94%) and/or full- or part-time employees (26%). Data collection occurred between November 2017 and April 2018.

### Stimulus material

The audiovisual stimuli were designed to resemble public service announcements (PSA) addressing work-related stress, a topic chosen due to being an important factor of peoples’ overall stress levels and considered to be a major physical and psychological threat [43]. Perceived job stress has increased, especially in recent years [44], largely driven by technological advances that have changed how people live and work. For instance, mobile devices now allow employees to work anywhere at all times, which has given way to a rise in remote offices and working outside regular office hours. As a consequence, boundaries between work and personal life have continued to dissolve [45, 46], which can adversely affect workers and prompt their perceptions of stress and work overload. Prolonged periods of high stress have been found to be especially harmful for individuals’ physical and mental health [43]. In turn, stressed employees are less efficient in completing tasks, which subsequently affects the economic situation of companies and, on an aggregated level, can impact the performance of entire countries [47]. Owing to such trends, the direct and indirect costs associated with stress and mental health have continued to rise [48]. Given all of the above, work stress is clearly a current, relevant health topic that needs to be addressed in public health communication.

In response, the PSAs designed for our study portrayed situations in which a person confronts demands and pressure at work that challenge their knowledge, capabilities, or resources. Following the common structure of fear appeals [5] and by operationalizing emotional flow [14], the video clips were designed to contain two distinct parts. The content of the video as well as the elements were the same for all experimental groups. Emotional valence was manipulated with different emotional framings in the second part of the PSA. In the first part, all clips aimed at inducing fear, which was achieved by applying threatening cues, namely by displaying situations in which a character experiences intense forms of work-related stress (e.g., getting called by a supervisor outside regular office hours or confronting an unsurmountable amount of work without any chance of ever being completed). The formal design of the PSAs, both visually and acoustically, sought to emphasize the negative and threatening aspects of work-related stress. Thus, the goal of the first part of the video, held constant for all versions of the stimulus, was to present stress as a potential risk to individuals’ health and thus promote the perception of a threat.

The second part of the video, by contrast, provided information about how to overcome or cope with stress in order to avoid the mentioned negative outcomes. To that purpose, the videos cited a goal (“Avoid stress—prevent stress”) and gave specific behavioral advice on how to achieve it. In accordance with recommendations published by the World Health Organization [49], the suggested behaviors included getting sufficient sleep, regularly engaging in physical activity, taking regular breaks, denying requests that would trigger unnecessary stress, and feeling free to occasionally make oneself unavailable (i.e., silencing one’s phone). All of those recommendations aim to reduce or avoid stress and help people to relax or recover. Each piece of advice was depicted visually (e.g., the character silence their mobile phone) and additionally emphasized by providing the recommendations as subtitles. Altogether, the second part of the video constituted the efficacy-fostering element of the message.

The second part of each stimulus video, containing the efficacy information, was manipulated to evoke different valence shifts. In particular, we created two groups of stimuli and manipulated the emotional framing in order to evoke different emotional flows by altering the audiovisual presentation (e.g., background music and formal image parameters). One group of stimuli was supposed to keep the audience in the same negative valence during exposure, starting with evoking fear in the first part while the second part featured, for example, frightening music and dark imagery to retain the negative valence. The other group of stimuli were the same in the first part but then introduced joyous music and bright colors in the second part, which was supposed to result in a shift to positive valence.

To increase the external validity, the manipulated video clip (length: approximately 2 minutes long) was integrated into a commercial break, embedded in a health-related TV show.

### Measures

The questionnaire assessed the constructs of the proposed path model based on the EPPM and gathered participants’ sociodemographic data and information about their personality traits.

#### Emotional flow and shifts

Experiences of emotional valence during exposure to the videos were measured with CRM (1 Hz [50]). Participants used a vertical slider on a tablet to continuously indicate the emotional valence from 0 (*unpleasant*) to 100 (*pleasant*). Such data served to provide spontaneous, cognitively unbiased insights into the dynamic evolution of emotional experiences during exposure to a message. At the same time, it served as a stimulus evaluation check for applied manipulations of shifts in valence across different versions of the stimulus. Later, the difference between the average emotional experience before and after the shift was calculated as an indicator of the emotional shift experienced (*M* = 7.46, *SD* = 16.01). For those values, positive means indicate a shift toward a positive valence for all groups on average. Because the second part of the videos focused on efficacy information, not the threat, it typically generated positive feelings. For the analysis, a *z*-standardized variable was created (min. = -2.64, max. = 3.70) to facilitate the interpretation of the results and compensate for individual differences in the range of using the slider. “Experienced shift” hereafter refers to the observed changes from the continuous response measurement.

#### Efficacy perceptions

Efficacy perceptions were measured with the Risk Behavior Diagnosis scale [51], which gauged participants’ perceptions of self-efficacy and response efficacy with respect to the presented recommendations in the PSA (e.g., “If I apply the measures recommended in the PSA, I will feel less stressed” or “The recommended measures are effective to reduce stress”). Responses to three items were given on a 5-point Likert-type scale ranging from 1 (*do not agree at all*) to 5 (*fully agree*). An efficacy index was constructed from calculating the average of the three items such that higher values indicated stronger efficacy perceptions (*M* = 3.79, *SD* = 0.75, α = .74).

#### Behavioral intentions

To assess behavioral intentions, participants were asked to rate how likely (1 = *very unlikely*, 5 = *very likely*) they were to follow the messages’ recommendations (see “Stimulus Material” for an overview of recommended behaviors) on a single item (*M* = 3.06, *SD* = 1.04 [52]).

#### Personal stress

To measure participants’ levels of stress, the Trier Inventory for Chronic Stress (TICS) scale [53] was administered, on which participants were asked to rate how often (1 = *never*, 5 = *very often*) they encounter specific stressful situations in their everyday lives. A stress index was constructed from calculating the mean of the 22 items (*M* = 3.00, *SD* = 0.63, α = .91).

### Data analysis

Before conducting the path analysis, zero-order correlations were examined (Table 1). Data were analyzed with PROCESS (V3.5 [54]) for IBM SPSS statistics (custom model with two mediators and a moderator; see Fig 1). The 95% confidence intervals were based on 10,000 bootstrapping samples. The experimental condition (i.e., valence shift) was entered as the independent variable, dummy coded to indicate no shift in valence or negative to negative as 0 and shift in valence or negative to positive as 1. Indices of the experienced shift (i.e., continuous response measure) and perceived efficacy were included as mediators. Stress was mean-centered and introduced as a moderator between the mediators. Behavioral intention served as the dependent variable. All regression coefficients (*B*) presented herein are unstandardized. Like most regression programs, PROCESS uses listwise deletion of missing data [54]. Consequently, the following analysis is based on data of 222 participants.

**Table 1:** Pearson zero-order correlations.

Variable	1	2	3	4
1 Valence shift	–			
2 Experienced shift^a^	.37**	–		
3 Perceived efficacy	-.02	.09	–	
4 Stress^b^	-.04	.14*	-.05	–
5 Behavioral intentions	.05	.02	.52**	-.04

*N* = 252; *n*’s due to missing values:

^a^*n* = 230

^b^*n* = 243

*p < .05

**p < .01.

## Results

### Manipulation check

**Fig 2:**
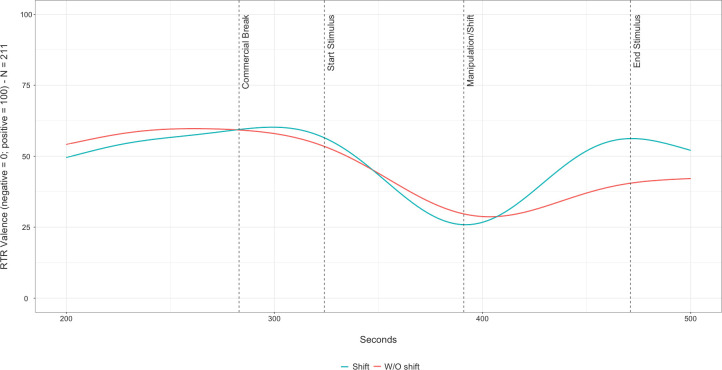
Subjective indication of valence (Pleasant/unpleasant) of the two Stimuli Groups.

Means of the data collected with CRM were calculated for every second during exposure within each experimental group. Such data clearly indicated that the intended shifts in the stimulus material triggered shifts in the valence experienced (Fig 2). Participants’ indication of valence did not differ during the first part of the video (i.e., threatening cues by showing the protagonist suffering from stress); those depictions consistently triggered unpleasant feelings across all groups. Subsequently, during the second part, participants’ experiences clearly fluctuated in valence as the manipulations changed; positive versions of the video prompted higher indications of pleasantness than the negative ones. As assumed, a slight shift toward a positive valence for the groups with negative emotional framing also occurred, because efficacy information alone can generate positive perceptions and even trigger feelings of relief [14].

Furthermore, an unpaired *t* test demonstrated statistically significant differences between the experimental groups. In particular, participants who watched a video without a valence shift indicated weaker shifts (*M* = 2.44, *SD* = 13.46) than participants who watched one with a shift from negative to positive valence, *M* = 14.50, *SD* = 16.70, *t*(182) = -5.941, *p* < .001.

### Path analysis

**Fig 3:**
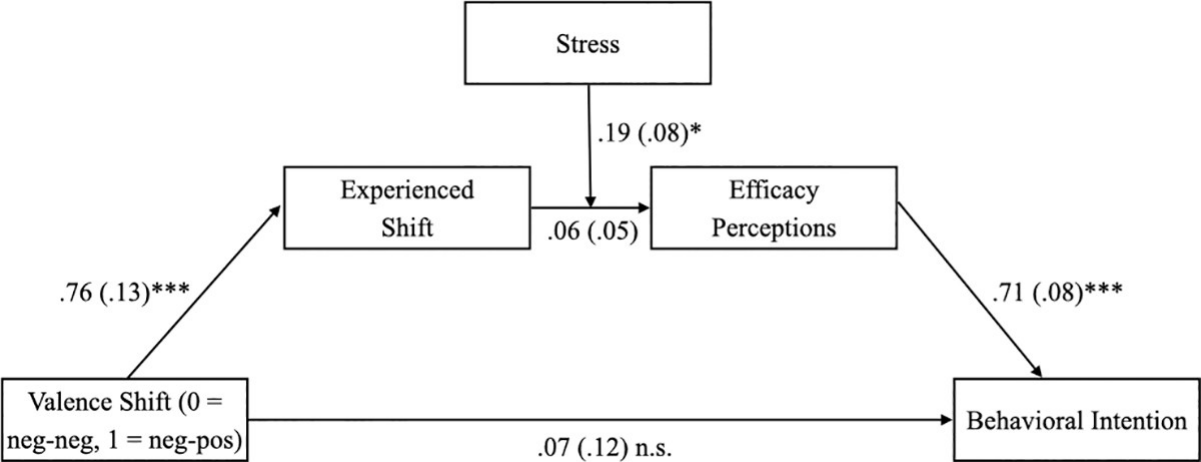
Unstandardized coefficients for moderation of mediation effect of stress. Standard errors are shown in parentheses. Indirect effect stress -.65: *B* = -.03, *SE* = .04, 95% CI [-.11, .03]. Indirect effect stress -.01: *B* = .03, *SE* = .03, 95% CI [-.02, .09]. Indirect effect stress .55: *B* = .09, *SE* = .04, 95% CI [.02, .17]. Index of moderated mediation: *B =* .10, *SE* = .05, 95% CI [.03, .21]. * p < .05. ** p < .01. *** p < .001.

The successful manipulation of valence in the second part of the videos was also reflected in the path model. Relative to ones who watched videos without a shift in valence, participants exposed to a shift from negative to positive indicated a stronger positive shift (*B* = .76, *SE* = .13, *p* < .001; H1 confirmed). Beyond that, results did not confirm a relationship between a larger upward shift in valence and efficacy perceptions (*B* = .06, *SE* = .05, *p* = .206; H2 rejected). This regression coefficient estimates the effect of the experienced shift when the variable stress is zero—that is, a medium level of stress [54]. In line with H3, the general personal stress level, indicated by the TICS scale, positively moderated the relationship between the valence shift experienced and efficacy perceptions (*B* = .19, *SE* = .08, *p* = .018). Given higher levels of stress, a positive shift in the second part of the message seemed to promote participants’ efficacy perceptions. The perceptions of participants with a (below-)average level of personal stress were not affected by (experiencing) a positive shift. As hypothesized, more pronounced perceptions of efficacy increased intentions to follow the messages’ recommendations (*B* = .71, *SE* = .08, *p* < .001; H4 confirmed). The results also support the notion that cognitive processes are crucial to a message’s effectiveness. In particular, data did not indicate any direct effect of the shift in valence (i.e., experimental condition) on behavioral intentions (*B* = .07, *SE* = .12; *p* = .577; H5 confirmed). Also, as expected, messages with a positive shift in valence indirectly improved participants’ reported likelihood of following the message’s recommendations, mediated by their experience with the shift and efficacy perception. However, that effect occurred only for participants with high levels of general stress, *B* = .09, *SE* = .04, 95% CI [.02, .17], but not for those with lower levels of stress, *B* = -.03, *SE* = .04, 95% CI [-.11, .03], which confirmed H6 (Fig 3, Tables 2 and 3).

**Table 2:** Results of moderated mediation.

	Outcomes											
	M1: Experienced Shift ^a^				M2: Perceived Efficacy ^b^				Y: Behavioral Intention ^c^			
	*B*	*SE B*	*p*	95% *CI* [LL, UL]	*B*	*SE B*	*p*	95% *CI* [LL, UL]	*B*	*SE B*	*p*	95% *CI* [LL, UL]
Valence Shift	.76	.13	< .001	.50, 1.01					.07	.12	.577	-.17, .31
Experienced Shift					.06	.05	.206	-.03, .16				
Stress					-.10	.08	.212	-.26, .06				
Experienced Shift x Stress					.19	.08	.018	.03, .35				
Perceived Efficacy									.71	.08	< .001	.55, .87

Summary for effects of valence shift on behavioral intention mediated by experienced valence shift and efficacy perceptions and accounting for the moderating effect of stress on the relationship between experienced valence shift and efficacy perceptions.

^a^*R*^2^ = .13 (*p* < .001).

^b^*R*^2^ = .04 (*p* = .034).

^c^*R*^2^ = .26 (*p* < .001).

Indirect effect Stress -.65: *B* = -.03, *SE* = .04, 95% *CI* [-.11, .03]. Indirect effect stress -.01: *B* = .03, *SE* = .03, 95% *CI* [-.02, .09]. Indirect effect stress .55: *B* = .09, *SE* = .04, 95% *CI* [.02, .17]. Index of moderated mediation: *B =* .10, *SE* = .05, 95% *CI* [.03, .21].

**Table 3:** Conditional effects of experienced shift on perceived efficacy by stress.

	Outcome			
	*Perceived Efficacy*			
Stress (mean centered)	*B*	*SE B*	*p*	95% *CI* [LL, UL]
Low (-.65)	-.06	.07	.420	-.21, .09
Middle (-.01)	.06	.05	.222	-.04, .16
High (.55)	.17	.06	.009	.04, .29

## Discussion

Research on fear appeals has generally supported the notion that messages that succeed in promoting perceptions of threat while at once increasing efficacy perceptions encourage desirable health behaviors, in a combination referred to as a magic cell [31–33]. Consequently, fear appeals should first emphasize the risks of a health topic, thereby promoting perceptions of a threat and evoking emotional responses of fear. However, efficacy cues subsequently presented are likely to evoke other, more positive emotional states. Albeit theoretically sound, empirical research has thus far overlooked the consequences of such shifts in emotional responses during persuasive attempts (for exceptions, see [19, 38]).

Therefore, the aim of our study was to explicitly model and test the effects of those valence shifts during exposure to persuasive health messages. In particular, we examined the influence of shifts in valence—not only formally in the presented information but also as experienced by participants—within a fear appeal on the processing of efficacy cues as well as behavioral intentions with respect to the recommendations provided. That investigation was performed with the threat of work-related stress, a health-related topic with particular relevance for workers today. CRM data were gathered during exposure to the PSAs in order to obtain a continuous indicator of participants’ perceptions of valence and verify their subjective experience of the valence shift introduced. According to data collected with CRM, the manipulations succeeded in eliciting different emotional flows in accordance with the intended directions, which allowed mapping the evolution of valence experiences during exposure to the message and provided proof of the existence of emotional flows.

Data further indicated that fear appeals starting with a threat but shifting to positively framed information about efficacy triggered larger positive shifts in valence than appeals with negatively framed efficacy cues. In turn, those positive shifts fostered efficacy perceptions, although only for people to whom the health issue was relevant. Thus, a positively experienced shift in valence only fostered efficacy perceptions when participants reported a higher level of stress in their personal lives. Those results, aligning with earlier findings, provide additional evidence of how the relevance of an issue and one’s personal involvement with it factor into message processing and outcomes [e.g., 22, 39–41]. Ultimately, the pronounced efficacy perception correlated positively with stronger intentions to follow the messages’ recommendations for proactively managing work-related stress. That result also confirmed previous findings about the importance of efficacy perceptions to the effectiveness of persuasive health information [32].

Our findings support the results of other studies illustrating that messages that shift in valence are more effective than unvarying ones [e.g., 20, 37]. By extension, they suggest that threatening cues focused only on evoking fear risk their effectiveness compared with more creative solutions that integrate emotional shifts into their narratives. If so, then the effectiveness of persuasive messages can be improved by framing efficacy information with positive emotions, because a positive emotional shift serves as a safety cue or buffer against how the negative emotional experience (i.e., fear) is absorbed [21].

Taken together, our study’s results empirically support Nabi’s [14] argument that a valence shift in emotional experience within health-related messages can bolster their impact. As such, they provide experts working in health promotion and disease prevention with a means to create more effective messages, namely “by presenting information within carefully ordered, emotionally evocative sequences” (p. 120). According to our results, efficacy cues charged with positive emotions heighten audiences’ intention to adapt and implement recommendations about preventing and coping with work-related stress (e.g., sleeping more and silencing one’s phone). Such significant interaction with participants’ individual stress levels indicates that personal relevance is an important prerequisite for shifts in fear appeals to exert any effect.

## Limitations and future research

For our research, we adapted the widely studied threat-then-efficacy pattern of fear appeals [5]. Despite results suggesting that introducing emotional shifts in valence seems to improve the effectiveness of messages, our study did not involve examining the effects of messages beginning with positively valenced information followed by either another positively or a negatively valenced part of the message. Such messages might produce different results. For instance, examining the effects of messages beginning with a positive valence versus messages beginning with a negative valence, Wang and Lang [55] found that cognitive effort toward an advertisement was higher when participants had previously been exposed to positively instead of negatively valenced content. However, due to our study’s design—that is, always beginning with a threat cue—it was impossible to contradict or confirm Wang and Lang’s [55] findings. Future research that accounts for and compares other combinations of shifts in valence would allow also investigating inter-emotional as well as intra-emotional differences with regard to their effectiveness.

Furthermore, our study did not account for arousal (i.e., intensity of physiological activation, ranging from calm to excited), despite its being another important dimension characterizing emotional experiences. Basing their argument on excitation transfer theory [56], Nabi and Green [57] assumed that the arousal of a preceding part of a mediated message (e.g., the threat component in our study) influences the subsequent experience during exposure. In turn, that dynamic could prompt a more intense post-shift emotional experience that could affect the processing of the information presented afterward (e.g., the variously framed efficacy cues in our study). To investigate that process, future studies should seek to account for the arousal dimension of emotional experiences along with valence. Physiological indicators such as electrodermal activity [58] or the arousal dimension of the self-assessment manikin [59] could supply additional evidence about cognitive and emotional processing during exposure to messages. Beyond that, Nabi [14] has assumed that emotional shifts may foster the allocation of cognitive resources, which could manifest in orienting responses at a physiological level [58]. Such physiological data could thus confirm that cognitive processing is more pronounced after a shift than a non-shift. The data could also indicate the possible effectiveness of placing information central to messages around those shifts. However, empirical evidence about that possibility remains pending.

Calculating the difference between the average emotional experience before and after the shift as an indicator of the valence shift experienced comes with the disadvantage of partly losing the dynamic nature of the measurement. Nevertheless, by following this approach we are able to accurately capture the (valence) experiences of our participants during exposure while recency effects or memory effects usually bias post exposure measures. This allows to collect significantly less biased information on the extent and direction of an experienced shift from negative to positive and to draw more accurate conclusions about the effect of experienced shifts on health-relevant outcomes. Another option to capture a shift would have been to stop the video several times and to ask participants about their feelings at every stopping cue. However, this would have interrupted the flow during message exposure. Even though moving a slider to indicate momentary emotional states in the CR measurement may also disturb the experience of the stimuli, the measure is less intrusive than stopping the video several times. In our study, participants indicated that using the slider was not disruptive (33%) or not disruptive at all (39%). Using CRM, we were able to measure valence experienced during exposure without being unduly intrusive and without interrupting the flow of exposure to the messages, which might be even of more significance and beneficial for longer and more complex stimuli.

## Conclusion

Our study has provided initial empirical insights into the dynamic evolution of emotional experience (i.e., emotional flow) during exposure to health messages containing different emotional cues. As such, it has confirmed the assumption that inducing a valence shift can improve the effectiveness of communicative measures for health promotion and disease prevention. More concretely, the data indicated that experiencing a shift from negative to positive valence during message exposure, as indexed by CRM, can particularly benefit people affected by higher levels of stress, for it increases their perceptions of efficacy. Consequently, and in line with previous studies, perceiving a health risk as manageable and counteractive measures as effective increases behavioral intentions to perform the recommended behavior.
